# Genetic Implication of Specific Glutamatergic Neurons of the Prefrontal Cortex in the Pathophysiology of Schizophrenia

**DOI:** 10.1016/j.bpsgos.2024.100345

**Published:** 2024-06-08

**Authors:** Claire E. Tume, Sophie L. Chick, Peter A. Holmans, Elliott Rees, Michael C. O’Donovan, Darren Cameron, Nicholas J. Bray

**Affiliations:** a Centre for Neuropsychiatric Genetics & Genomics, Division of Psychological Medicine & Clinical Neurosciences, Cardiff University, Cardiff, Wales, United Kingdom; b Neuroscience & Mental Health Innovation Institute, Cardiff University, Cardiff, Wales, United Kingdom

**Keywords:** Gene expression, Genetic, Genome-wide association study (GWAS), Prefrontal cortex, Rare variant, Schizophrenia

## Abstract

**Background:**

The prefrontal cortex (PFC) has been strongly implicated in the pathophysiology of schizophrenia. Here, we combined high-resolution single-nuclei RNA sequencing data from the human PFC with large-scale genomic data for schizophrenia to identify constituent cell populations likely to mediate genetic liability to the disorder.

**Methods:**

Gene expression specificity values were calculated from a single-nuclei RNA sequencing dataset comprising 84 cell populations from the human PFC, spanning gestation to adulthood. Enrichment of schizophrenia common variant liability and burden of rare protein-truncating coding variants were tested in genes with high expression specificity for each cell type. We also explored schizophrenia common variant associations in relation to gene expression across the developmental trajectory of implicated neurons.

**Results:**

Common risk variation for schizophrenia was prominently enriched in genes with high expression specificity for a population of mature layer 4 glutamatergic neurons emerging in infancy. Common variant liability to schizophrenia increased along the developmental trajectory of this neuronal population. Fine-mapped genes at schizophrenia genome-wide association study risk loci had significantly higher expression specificity than other genes in these neurons and in a population of layer 5/6 glutamatergic neurons. People with schizophrenia had a higher rate of rare protein-truncating coding variants in genes expressed by cells of the PFC than control individuals, but no cell population was significantly enriched above this background rate.

**Conclusions:**

We identified a population of layer 4 glutamatergic PFC neurons likely to be particularly affected by common variant genetic risk for schizophrenia, which may contribute to disturbances in thalamocortical connectivity in the condition.

## Plain Language Summary

The prefrontal cortex (PFC) has been strongly implicated in the underlying biology of schizophrenia. We tested whether specific cell populations within the PFC preferentially express genes that increase risk for the disorder. We found that a particular type of PFC neuron prominently expresses genes associated with schizophrenia, suggesting its involvement in the condition.

The prefrontal cortex (PFC) is known to play an important role in executive functions such as working memory, attention, and decision making (1). These functions are typically impaired in people with schizophrenia (2), who show reduced activation of the PFC during such tasks (3). Functional neuroimaging studies have also yielded evidence for decreased functional connectivity between the PFC and other brain regions in schizophrenia, including the hippocampus (4,5) and thalamus (6,7). Reductions in PFC volume have been reported in first-episode schizophrenia (8) and in people at high genetic risk for the disorder (9).

Large-scale genomic studies have identified hundreds of genetic variants associated with schizophrenia, including alleles that are common in the general population (10) as well as rare variations in protein-coding sequence (11). Notably, both types of risk variation are enriched in genes with high expression specificity for the PFC (10,11). It is now possible to measure global gene expression in individual cell populations from complex tissues using single-cell (or single-nuclei) RNA sequencing (sc/snRNA-Seq). By combining these data with genomic data for a given disorder, specific cell populations that are likely to mediate genetic associations with that condition can be identified (12, 13, 14). Using this approach, we recently reported enrichment of schizophrenia common variant liability in genes with pronounced expression in immature neuronal populations of the early second trimester fetal brain, with the most significant enrichment in developing glutamatergic neurons of the frontal cortex (15). Enrichment of schizophrenia risk variation has also been observed in genes with high expression specificity for mature neuronal populations of the adult human PFC (12,16,17). However, the PFC is known to undergo important developmental changes from birth until early adulthood that are of potential relevance to schizophrenia pathogenesis (18), but which may not have been adequately captured in these previous studies. In the current study, we combined large-scale genomic data for schizophrenia with snRNA-Seq data from the human PFC spanning midgestation to adulthood (19) to strongly implicate a specific population of layer 4 glutamatergic neurons in genetic risk for the disorder.

## Methods and Materials

### snRNA-Seq Data

snRNA-Seq data from the study of Herring *et al.* (19) were downloaded from https://storage.googleapis.com/neuro-dev/Processed_data/RNA-all_full-counts-and-downsampled-CPM.h5ad. Data were derived from a total of 154,748 nuclei from the PFC of 26 individuals ranging in age from gestation week 22 to 40 years. For a full description of samples and data generation, see the original publication (19). Initially, we analyzed the 86 cell populations identified across all developmental stages. For downstream processing, the count matrix and meta data were extracted from the h5ad file and converted into a Seurat object using Seurat version 4.3.0 (20).

### Calculation of Cell Expression Specificity Scores

Cell specificity scores for each gene were calculated using the EWCE R package, version 1.9.2 (21). Before this, 2 cell populations, Oligo-6 (number of cells = 7) and Oligo-7 (number of cells = 3), were removed because they had too few cells to capture representative measures of gene expression, leaving 84 cell populations for analysis. Nonautosomal genes, which could introduce sex-related donor bias, and genes in the extended major histocompatibility complex region on chromosome 6 (hg19 coordinates: chr6 start = 25000000; end = 35000000), a region of extensive linkage disequilibrium (LD), were removed prior to calculating specificity scores so that they would not be included in subsequent enrichment analyses. We then used EWCE (21) to normalize the unique molecular identifier counts [using scTransform (22)], remove uninformative (e.g., low or sporadically expressed) genes, and generate a CellTypeDataset object. The latter contains a matrix of expression specificity scores for each gene in each cell type, which were calculated by dividing a gene’s mean expression in a single cell type by the sum of that gene’s mean expression in all cell types. The expression specificity scores of all genes in each cell population are provided in Table S1 in Supplement 2.

### Testing Enrichment of Schizophrenia Common Variant Liability in Cell Populations of the PFC

We used MAGMA (23) and stratified LD score regression (SLDSR) (13) to test for enrichment of schizophrenia common variant liability in gene sets representing the top expression specificity decile for each cell population, as performed previously (10,14,15). Genome-wide association study (GWAS) summary statistics were taken from participants of European ancestry (53,386 people with schizophrenia and 77,258 control participants) in the largest GWAS of the disorder conducted to date (10). GWAS data for human height (approximately 700,000 individuals of European ancestry) (24) were used as a comparison nonbrain phenotype, and GWASs of attention-deficit/hyperactivity disorder (>38,000 cases and >186,000 controls) (25), autism (>18,000 cases and >27,000 controls) (26), major depressive disorder (>135,000 cases and >344,000 controls) (27), bipolar disorder (>41,000 cases and >371,000 controls) (28), and neuroticism (>313,000 individuals of European ancestry) (29) were used as comparison brain-related phenotypes. Links for the GWAS data used in this study are provided in Table S2 in Supplement 2. The Ensembl GRCh37 (https://grch37.ensembl.org) (30) assembly was used as the reference for gene locations, with patches, duplicate genes, genes within the extended major histocompatibility complex region, and nonautosomal genes removed. For the primary analyses, we included all genes (noncoding and protein-coding) in the top decile of expression specificity for each cell type (provided in Table S3 in Supplement 2). We also tested only protein-coding genes for consistency with earlier studies (10,14,15) and only noncoding genes to assess their potential contribution to observed enrichments (by filtering the gene biotype category for and against protein_coding, respectively).

MAGMA gene set enrichment analyses (23) of common variant associations were performed on genes in the top specificity decile of each cell population using the single nucleotide polymorphism (SNP)–wise mean model. Gene boundaries were extended by 35 kb upstream and 10 kb downstream of the transcription start and end site, respectively, as performed previously (10,14). SNP *p* values from GWAS summary statistics were averaged across SNPs located within these gene boundaries using the 1000 Genomes Project phase 3 data (31) as the reference for LD. SLDSR (13) was performed to test the enrichment of schizophrenia SNP heritability in genes in the top specificity decile of each cell population. Gene boundaries were extended by the recommended length of 100 kb upstream and downstream of the transcribed regions (13). LD scores were estimated for each SNP in relation to neighboring SNPs within 1 cM using the 1000 Genomes Project phase 3 reference panel files to approximate LD. Schizophrenia SNP heritability was then ranked for each gene set using a joint fit model that accounts for SNP heritability due to general genomic annotations, such as enhancers and conserved regions. To determine significance, a *z* score was calculated based on whether schizophrenia SNP heritability was greater in each gene set versus these 53 baseline model annotations (baseline model version 1.2) (13). All enrichment *p* values were one tailed. We highlight cell enrichments that met the Bonferroni threshold for significance in both MAGMA and SLDSR after correcting for 84 tested cell populations (*p* < 5.95 × 10^−4^). To assess the robustness of our results, we also tested the top 2000 most specific genes for each cell population (8.4%–16.3% of all genes detected in each population, mean = 9.6%).

### Developmental Trajectory Enrichment Analyses

To test enrichment of schizophrenia common variant liability in cells across the developmental trajectory of L4-RORB-LRRK1 neurons, we used a method developed by Shulman and Elkon (https://github.com/ElkonLab/scGWAS) (32). Briefly, cells annotated to L4-RORB-LRRK1 neurons and their developing neuronal precursors were isolated from the Seurat object, and the filtered count matrix was extracted. Genes expressed by <10 cells were removed and the data subsequently log-normalized. A schizophrenia cell-trait association score was then calculated for each cell by running separate instances of a MAGMA gene property analysis. Here, schizophrenia-associated gene-wide *p* values (calculated using the MAGMA SNP-wise mean model) for each gene expressed by each single cell were regressed against the log-normalized expression values of those genes in the cell (accounting for the average normalized expression of each gene in the dataset). To associate the schizophrenia cell-trait association scores to the L4-RORB-LRRK1 developmental trajectory, pseudotime analysis was first run using Monocle 3 (33), and a single trajectory progressing from PN-dev to L4-RORB-LRRK1 cells was identified. We used these pseudotime values to test whether schizophrenia cell-trait association scores would increase with progression along the L4-RORB-LRRK1 developmental trajectory using a linear regression model, as implemented in the scGWAS pipeline (32).

### Gene Ontology Enrichment Analyses

To investigate biological processes that potentially mediate enrichment of common variant liability for schizophrenia in the most strongly implicated cell population, we first performed Gene Ontology (GO) enrichment analyses on the genes in the top expression specificity decile for the cell type using the GOTERM_BP_FAT category in the DAVID 2021 Bioinformatics Resource (34, 35) and a background list comprising all genes detected in that cell population. The online resource Revigo (http://revigo.irb.hr/) (36) was used to remove redundant GO terms. To identify the processes relevant to schizophrenia, we then used MAGMA and SLDSR to test enrichment of schizophrenia associations within genes in the top decile of expression specificity for the cell type that also belong to each of the 20 most significantly overrepresented GO terms.

### Expression Specificity of Fine-Mapped Genes for Schizophrenia

To identify cell populations with higher expression specificity for 67 autosomal genes prioritized within schizophrenia-associated loci by genetic fine-mapping (10), a 1-sided Wilcoxon rank-sum test was performed for each cell population. Genes not expressed by a cell population (with a specificity score of 0) were removed before running the test. The expression specificity score rankings of the fine-mapped genes were compared with the rankings of all other genes expressed in that cell type.

### Burden Analyses of Rare Protein-Truncating Coding Variants

Exome sequencing–derived rare coding variant data from a published Swedish schizophrenia case-control study (37) were obtained from the National Center for Biotechnology Information database of genotypes and phenotypes (dbGaP; accession number phs000473.v2.p2). Sample, variant, and genotype quality control for this dataset were previously performed using Hail (https://github.com/hail-is/hail) (38,39). We excluded one member of a duplicate (kinship coefficient > 0.354) or first-degree relative (kinship coefficient > 0.177) pair, individuals of non-European or Finnish ancestry, and samples with low sequencing coverage (mean genotype depth < 20×), which left 4079 schizophrenia cases and 5712 controls for rare coding variant analysis. Genotypes were excluded if they did not meet the following criteria: depth ≥ 10×, genotype quality score ≥ 30, allele balance ≤ 0.1 and ≥0.9 for homozygous genotypes for the reference and alternative allele, respectively; and allele balance between 0.25 and 0.75 for heterozygous genotypes. Variants were then excluded if they failed GATK VQSR filters, had a call rate < 0.9, failed Hardy-Weinberg equilibrium exact tests (*p* < 1 × 10^−8^), or occurred in a region of low complexity (40).

Variants were annotated using Ensemble Variant Effect Predictor, version 96 (41). Rare protein-truncating variants (PTVs) were defined as stop-gain, frameshift, or splice donor/acceptor variants observed as singleton mutations (i.e., one heterozygous carrier among all cases and controls) and absent from individuals in the nonpsychiatric dataset of the Exome Aggregation Consortium (version 0.3). We did not use the gnomAD (Genome Aggregation Database) to filter variants based on allele frequency because it is not possible to obtain a version that is independent from the Swedish case-control sample.

To test for an excess burden of rare PTVs in schizophrenia cases compared with controls, a one-tailed Firth’s penalized-likelihood logistic regression model was used, correcting for the first 10 principal components derived from the sequencing data, the exome-wide burden of rare synonymous variants, sequencing platform, and sex. When testing specific cell populations of the PFC, we corrected for the burden of rare PTVs detected in all protein-coding genes expressed across cells of the PFC.

## Results

### Prominent Enrichment of Schizophrenia Common Variant Liability in Genes With High Expression Specificity for a Population of Layer 4 Glutamatergic Neurons of the PFC

**Figure 1:**
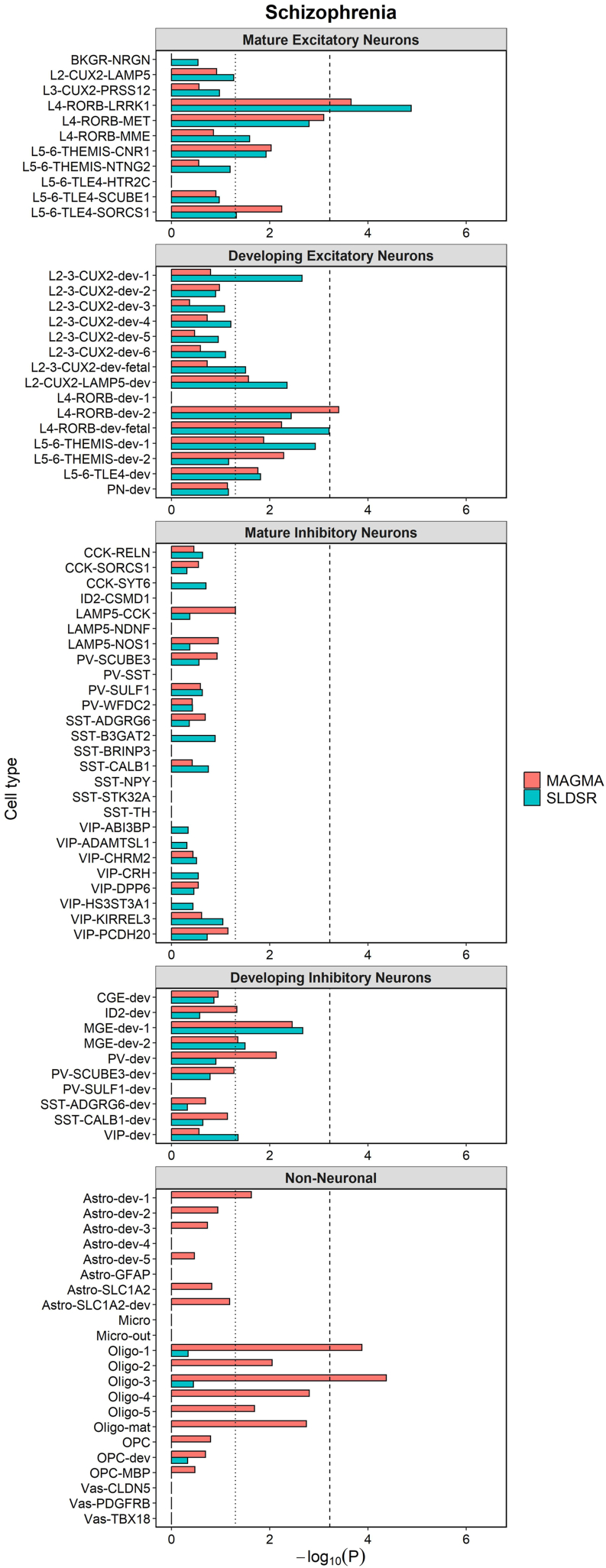
MAGMA and stratified linkage disequilibrium score regression (SLDSR) −log_₁₀_*p* values for enrichment of schizophrenia common variant liability within genes that are in the top decile of expression specificity for each cell population of the prefrontal cortex. The dotted line shows the nominal (*p* < .05) significance threshold, and the dashed line shows the Bonferroni-corrected *p* value threshold for the 84 cell populations tested (*p* < 5.95 × 10^−4^). Cell populations are labeled in accordance with Herring *et al.* (19) on the basis of cell markers. Astro, astrocyte; dev, developing cell; L, layer; Micro, microglia; Oligo, oligodendrocyte; OPC, oligodendrocyte precursor cell; Vas, vascular cell. For a full list of abbreviations, see Table S16 in Supplement 2.

Common variant liability to schizophrenia was enriched (*p* < .05, uncorrected) in both the MAGMA and SLDSR tests in genes with high expression specificity for 9 excitatory (i.e., glutamatergic) neuron populations and 2 developing inhibitory (i.e., GABAergic [gamma-aminobutyric acidergic]) neuron populations (Figure 1 and Table S4 in Supplement 2). Only enrichment in genes in the top decile of expression specificity for a mature excitatory neuron population (L4-RORB-LRRK1) reached the experiment-wide Bonferroni-corrected threshold of *p* < 5.95 × 10^−4^ in both tests. The same result was obtained when an equal number of genes was tested for each cell type (Figure S1 in Supplement 1). This neuronal population expresses the layer 4 cortical marker *RORB*, encoding the transcription factor retinoic acid-related orphan receptor beta.

Our analyses diverge from previous cell-type enrichment analyses of schizophrenia (10,12,14,15) in that we included noncoding as well as protein-coding genes. We based this decision on accumulating evidence for a role of noncoding RNA genes in brain functions of potential relevance to neuropsychiatric disorders (42). When we restricted our analyses to only protein-coding genes or only noncoding genes in the top decile of expression specificity for each cell type, the enrichment of schizophrenia common variant liability in L4-RORB-LRRK1 neurons, although exceeding *p* < .05, no longer met the Bonferroni-corrected *p*-value threshold in either analysis (Figure S2A, B in Supplement 1; Tables S5 and S6 in Supplement 2), suggesting that both gene types contribute to the signal in these neurons. Furthermore, in the analyses based only on noncoding genes in the top specificity decile for each cell type (Figure S2B in Supplement 1 and Table S6 in Supplement 2), schizophrenia common variant liability was enriched in a prenatal precursor of L4-RORB-LRRK1 neurons (L4-RORB-dev-fetal) at the Bonferroni *p*-value threshold in both MAGMA and SLDSR analyses.

### Enrichment of Common Variants Associated With Other Brain-Related Traits in Genes With High Expression Specificity for Layer 4 Glutamatergic Neurons

We compared the pattern of cellular enrichment observed for schizophrenia common variant genetic associations with that for 5 other brain-related traits (attention-deficit/hyperactivity disorder, autism, bipolar disorder, major depressive disorder, and neuroticism) and the nonbrain phenotype of height (Figures S3–S8 in Supplement 1 and Tables S7–S12 in Supplement 2). Like those associated with schizophrenia, common variants associated with the personality trait of neuroticism were enriched in genes with high expression specificity for L4-RORB-LRRK1 in both the MAGMA and SLDSR tests at the Bonferroni-corrected *p*-value threshold (Figure S3 in Supplement 1 and Table S7 in Supplement 2). Genes in the top expression specificity decile for L4-RORB-LRRK1 were also enriched for common variant liability to attention-deficit/hyperactivity disorder (Figure S4 in Supplement 1 and Table S8 in Supplement 2) and bipolar disorder (Figure S5 in Supplement 1 and Table S9 in Supplement 2) at the Bonferroni-corrected *p*-value threshold in MAGMA and enriched for common variant liability to major depressive disorder at nominal significance (*p* < .05) in the SLDSR test (Figure S6 in Supplement 1 and Table S10 in Supplement 2). In contrast, we found no evidence that common genetic variants associated with autism were enriched in genes with high specificity for L4-RORB-LRRK1 in either test, where beta and *z* scores were close to 0 (Figure S7 in Supplement 1 and Table S11 in Supplement 2). Common variants associated with height were not consistently enriched in genes in the top expression specificity decile of any neuronal population (Figure S8 in Supplement 1 and Table S12 in Supplement 2).

### Enrichment of Schizophrenia Common Variant Genetic Liability Across the Layer 4 Glutamatergic Neuron Developmental Trajectory

In the study of Herring *et al.* (19), L4-RORB-LRRK1 neurons were first prominently detectable in the human PFC at 3 months of age and were reported by the authors to mature in childhood. The complete dataset of 86 cell populations (84 assayed in this study) contains at least 2 RORB+ developmental precursors of L4-RORB-LRRK1 neurons: L4-RORB-dev-fetal (detected in the PFC of donors at 22 and 24 weeks of gestation) and L4-RORB-dev-2 (detected in the PFC of donors at 34 weeks of gestation and in donors at 1–4 postnatal months). A third RORB+ neuronal population (L4-RORB-dev-1) was detected at 22 weeks of gestation but remained a small population within the PFC across postnatal development, suggesting that it is not a direct precursor of L4-RORB-LRRK1 neurons. The relative proportions of the PFC layer 4 RORB+ neuronal populations across development are shown in Figure S9 in Supplement 1. Genes in the top expression specificity decile of each RORB+ population only partially overlapped, with a maximum Jaccard index between RORB+ cell types of 0.31 (Figure S10 in Supplement 1).

**Figure 2:**
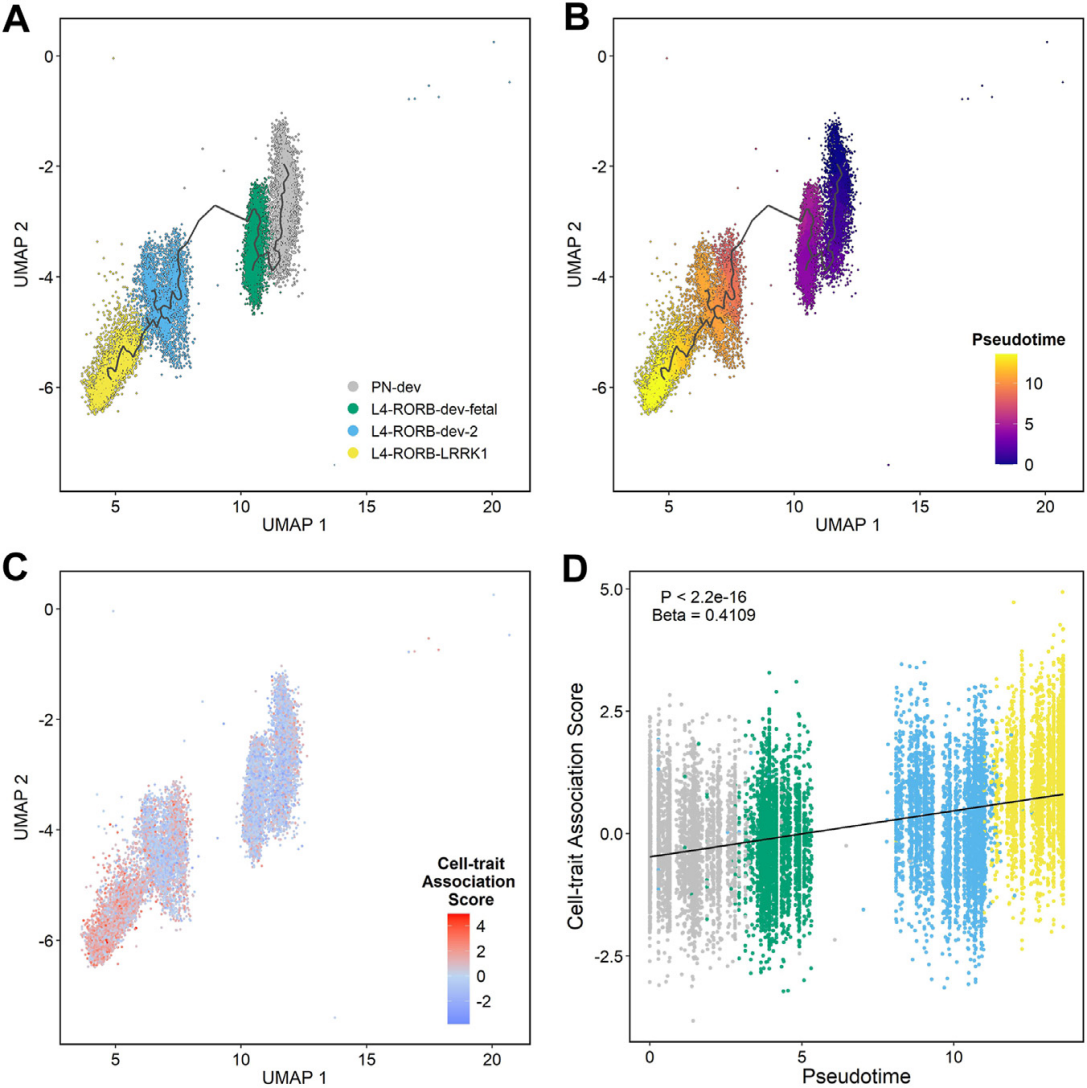
Pseudotime analyses of cells along the L4-RORB-LRRK1 developmental trajectory in relation to common variant genetic liability to schizophrenia. **(A, B)** Pseudotime analyses using Monocle 3 (33) ordered cells along a developmental trajectory from nascent principal neurons (PN-dev), through developing RORB+ neurons in fetal brain (L4-dev-fetal) and the neonatal period (L4-RORB-dev-2) to mature L4-RORB-LRRK1 neurons, as predicted by Herring *et al.* (19). **(C)** High cell-trait association scores (based on the relationship between MAGMA gene analysis schizophrenia *p* values and the expression level of each gene detected in that cell) were concentrated in cells annotated as L4-RORB-LRRK1 neurons. **(D)** Cell-trait association scores significantly increased along the L4-RORB-LRRK1 developmental trajectory (standardized beta = 0.41; *p* < 2.2 × 10^−16^).

We plotted the developmental trajectory of L4-RORB-LRRK1 neurons from developing glutamatergic principal neurons using pseudotime analysis (33) (Figure 2A, B). As a means of visualizing enrichment of schizophrenia common variant liability across this developmental trajectory, we applied the method of Shulman and Elkon (32), which generates a trait association score for each cell based on the relationship between MAGMA gene analysis *p* values for that trait and the expression level of every gene detected in that cell. Consistent with our analyses based on genes in the top decile of expression specificity for each defined cell population, cells with high cell-trait (schizophrenia) association scores were concentrated among those annotated as L4-RORB-LRRK1 neurons (Figure 2C), with increasing scores across the developmental trajectory (standardized beta = 0.41; *p* < 2.2 × 10^−16^) (Figure 2D). This pattern of enrichment was maintained in a series of leave-one-out tests, where cells belonging to a single donor were excluded from the analysis (Figure S11 in Supplement 1), indicating independence from donor effects.

### Biological Processes Driving Enrichment of Schizophrenia Common Variant Liability in Layer 4 Glutamatergic Neurons

**Figure 3:**
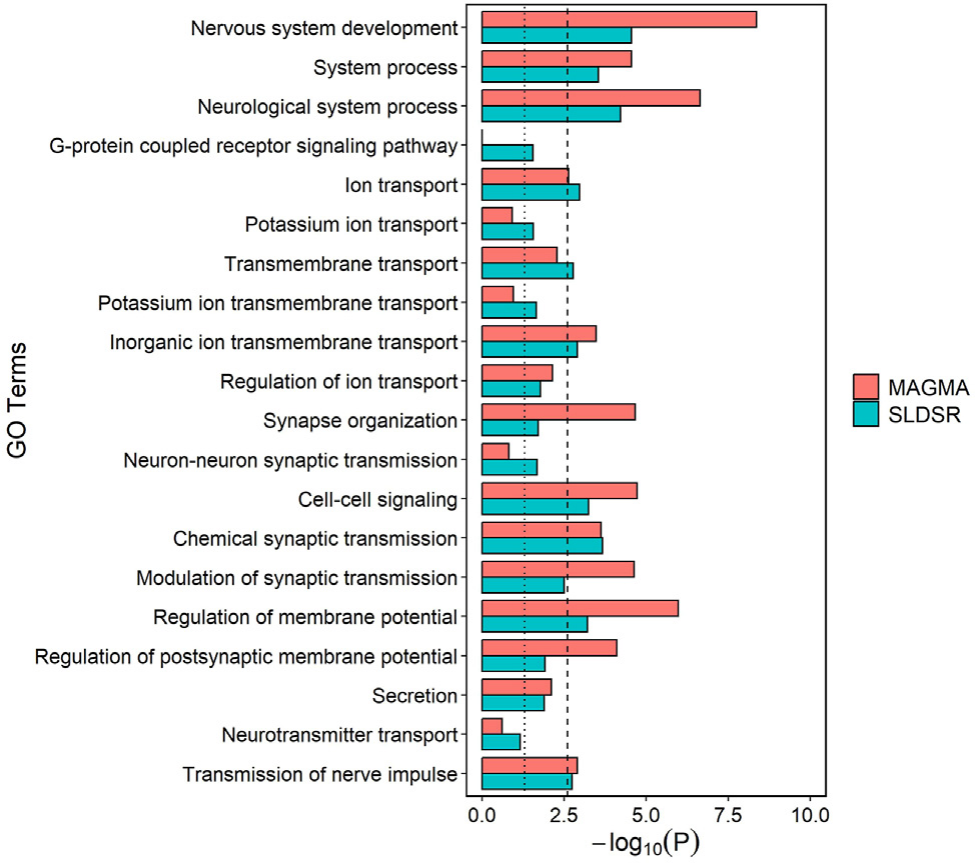
MAGMA and stratified linkage disequilibrium score regression (SLDSR) −log_₁₀_*p* values for enrichment of schizophrenia common variant liability in genes with high expression specificity for L4-RORB-LRRK1 neurons annotated to overrepresented Gene Ontology (GO) biological processes. The dotted line shows the nominal (*p* < .05) significance threshold, and the dashed line shows the Bonferroni-corrected *p* value threshold for the 20 GO terms that were tested (*p* < .0025).

Next, we explored biological processes that potentially underlie enrichment of schizophrenia common variant liability in L4-RORB-LRRK1 neurons. Initial GO analyses showed that genes in the top decile of expression specificity for this cell population were enriched for various terms related to neuronal development and function (Table S13 in Supplement 2). Then we tested enrichment of schizophrenia common variant associations in genes annotated to the 20 most significantly overrepresented GO terms belonging to the top decile of expression specificity for L4-RORB-LRRK1 neurons. Schizophrenia common variant liability was prominently enriched (at the Bonferroni-corrected threshold of *p* < .0025 in both the MAGMA and SLDSR tests) for genes related to nervous system development, ion transport, chemical synaptic transmission, and regulation of membrane potential (Figure 3).

### Fine-Mapped Genes for Schizophrenia Have Higher Expression Specificity for Glutamatergic Neuron Populations of the PFC

**Figure 4:**
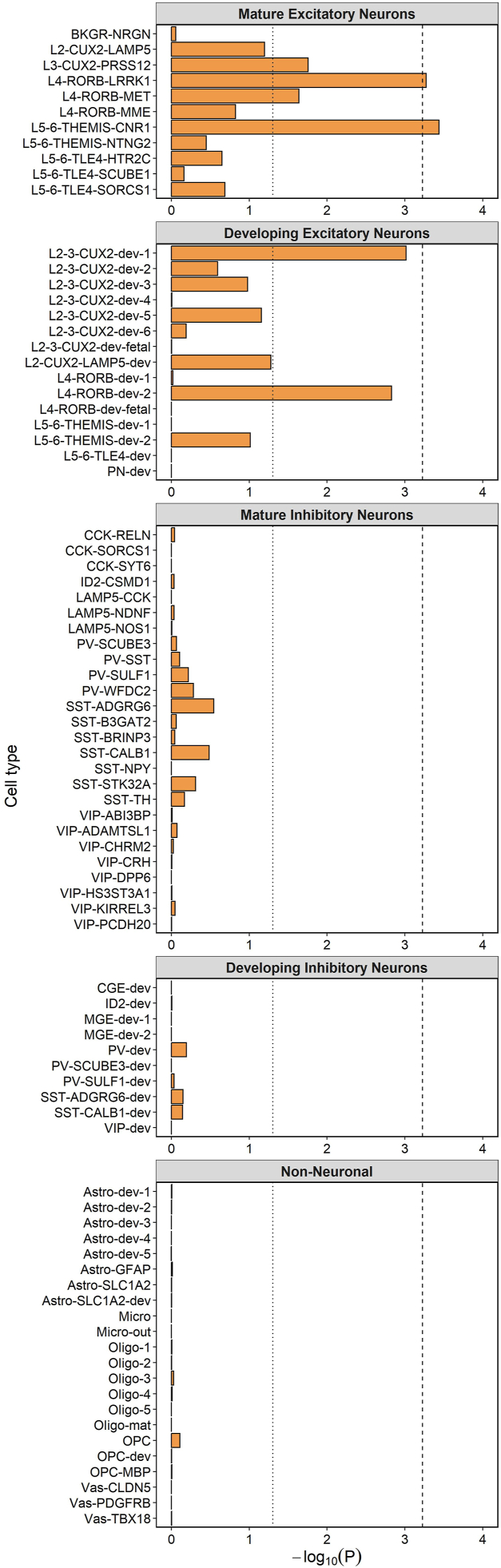
Wilcoxon rank-sum test −log_₁₀_*p* values for higher cellular expression specificity of 67 autosomal genes implicated in schizophrenia common variant susceptibility by genetic fine-mapping (10). The dotted line shows the nominal (*p* < .05) significance threshold, and the dashed line shows the Bonferroni-corrected *p* value threshold for the 84 cell populations tested (*p* < 5.95 × 10^−4^). Cell populations are labeled in accordance with Herring *et al.* (19) on the basis of cell markers. Astro, astrocyte; dev, developing cell; L, layer; Micro, microglia; Oligo, oligodendrocyte; OPC, oligodendrocyte precursor cell; Vas, vascular cell. For a full list of abbreviations, see Table S16 in Supplement 2.

Trubetskoy *et al.* (10) prioritized 70 genes at genome-wide significant risk loci for schizophrenia using genetic fine-mapping, of which 67 are autosomal. We investigated whether these genes had significantly higher specificity scores among those expressed in each defined cell population using one-sided Wilcoxon rank-sum tests. Fine-mapped genes for schizophrenia had significantly higher expression specificity (at the Bonferroni-corrected threshold) in L4-RORB-LRRK1 neurons and in a population of layer 5/6 glutamatergic neurons (L5-6-THEMIS-CNR1) (Figure 4). The expression specificity scores for all 67 fine-mapped genes in each cell type are provided in Table S14 in Supplement 2.

### Burden of Rare PTVs in Genes With High Expression Specificity for Cells of the PFC in Schizophrenia

Damaging rare coding variants are enriched in people with schizophrenia (37). Consistent with findings based on bulk RNA sequencing of the adult human PFC (11), we found an increased rate of rare PTVs in protein-coding genes expressed in at least one cell population of the human PFC in schizophrenia cases compared with controls (odds ratio = 1.08, *p* = 5.1 × 10^−6^). To identify cell populations of the PFC that may be particularly important for mediating effects of PTVs on risk for schizophrenia, we performed burden tests of rare PTVs in schizophrenia cases versus controls within protein-coding genes in the top decile of expression specificity for each cell type, while controlling for the background rate of rare PTVs in all protein-coding genes detected in the PFC (Table S15 in Supplement 2). Genes in the top specificity decile of 6 cell populations were enriched for PTVs in schizophrenia cases above the PFC background rate, including L4-RORB-dev-fetal and L4-RORB-dev-2, at *p* < .05; however, none of these associations survived correction for multiple testing.

## Discussion

Studies have shown that schizophrenia risk variation is enriched in genes with high expression specificity for neurons of the cerebral cortex (10,12, 13, 14, 15, 16, 17). However, to date, these studies have been limited to testing broad excitatory and inhibitory human neuron populations (e.g., 10, 12, 13, 14) or more fine-grained neuronal populations exclusively from the prenatal (15) or adult (16) human brain. In this study, we took advantage of a recent, high-resolution snRNA-Seq dataset of the human PFC spanning gestation to adulthood (19) to better define the neuronal populations that are likely to be functionally affected by genetic variation conferring susceptibility to the condition.

Genes with high expression specificity for a subpopulation of layer 4 glutamatergic neurons (L4-RORB-LRRK1) showed the most significant enrichment of common variant liability to schizophrenia. Herring *et al.* (19) reported that the maturation of these neurons increases during childhood, coinciding with a reduction of short-range intracortical connections and strengthening of long-range interregional connections (43). Unlike other regions of the cerebral cortex (e.g., the somatosensory cortex), neurons in layer 4 of the PFC seem to be specific to primates, and together with deep layer 3 neurons, they receive the primary, driving innervation from the thalamus (44). Several groups have reported reduced structural (6,45,46) and functional (6,7,47, 48, 49) connectivity between the thalamus and PFC in people with schizophrenia, a pattern also observed in their healthy first-degree relatives (50). Our findings suggest that genetic risk variation for schizophrenia could contribute to disruptions in thalamocortical connectivity by impairing the development and function of layer 4 excitatory neurons.

Contrary to our expectations based on reported disturbances in parvalbumin-positive (PV^+^) and somatostatin-positive PFC neurons in schizophrenia (51), we found no evidence for enrichment of common variant schizophrenia associations in genes with high expression specificity for any mature GABAergic cell population of the PFC. However, nominally significant enrichment of common variant associations was observed in genes with high expression specificity for developing GABAergic neuron populations, consistent with our previous analyses of the human ganglionic eminences (15,52). It has been reported that layer 4 PV^+^ neurons of the PFC receive a lower density of excitatory synapses in schizophrenia, with this predicting reduced messenger RNA expression of PV and the GABA synthesis enzyme GAD67 within these neurons (53). Therefore, it is plausible that observed reductions of these markers within layer 4 PV^+^ interneurons of the PFC in schizophrenia (51) partly reflect reduced synaptic input from the excitatory neuron population(s) that were more strongly implicated in common variant genetic liability to the disorder in the current study.

Our common variant analyses were based on SNPs mapping within the broad genomic coordinates of genes with high expression specificity for each defined cell type. Although this will capture risk alleles with effects on the expression, splicing, and amino acid sequence of the genes in which they are located, for some loci, the susceptibility gene(s) might have gone undetected due to long-range regulatory effects on distal genes. An alternative approach is to test enrichment of SNP heritability within potentially functional noncoding elements that have been mapped in individual cell populations [e.g., open chromatin profiling through single-nuclei transposase-accessible chromatin with sequencing (52, 54, 55, 56)]. However, the cell types implicated in complex traits through these different functional modalities have been shown to be highly consistent (56), and single-nuclei epigenomic datasets of the PFC (19) do not currently have the cellular resolution afforded by the snRNA-Seq data used in the current study.

Given its critical role in executive functions, the PFC is likely to be relevant to a range of neuropsychiatric phenotypes. We found some evidence that the layer 4 glutamatergic neuron population that we implicated in schizophrenia susceptibility is also associated with other brain-related traits, most convincingly the personality trait of neuroticism, with which schizophrenia is genetically correlated (57). The tables in Supplement 2 provide the specificity scores of genes expressed by 84 cell populations of the developing and adult PFC that can be used for further studies investigating the cellular basis of these and other traits.

## Supplementary Material

Supplement

Key Resources Table
