# Supplementary material for: Genetic Implication of Specific Glutamatergic Neurons of the Prefrontal Cortex in the Pathophysiology of Schizophrenia
Source: Biol Psychiatry Glob Open Sci. 2024 Jun 8;4(5):100345. doi: 10.1016/j.bpsgos.2024.100345 (PMC11295574; doi:10.1016/j.bpsgos.2024.100345)
Supplement: Supplement [file mmc1.pdf]

SUPPLEMENTARY INFORMATION

Genetic Implication of Specific Glutamatergic Neurons of the Prefrontal Cortex in the Pathophysiology of Schizophrenia

Tume *et al.*

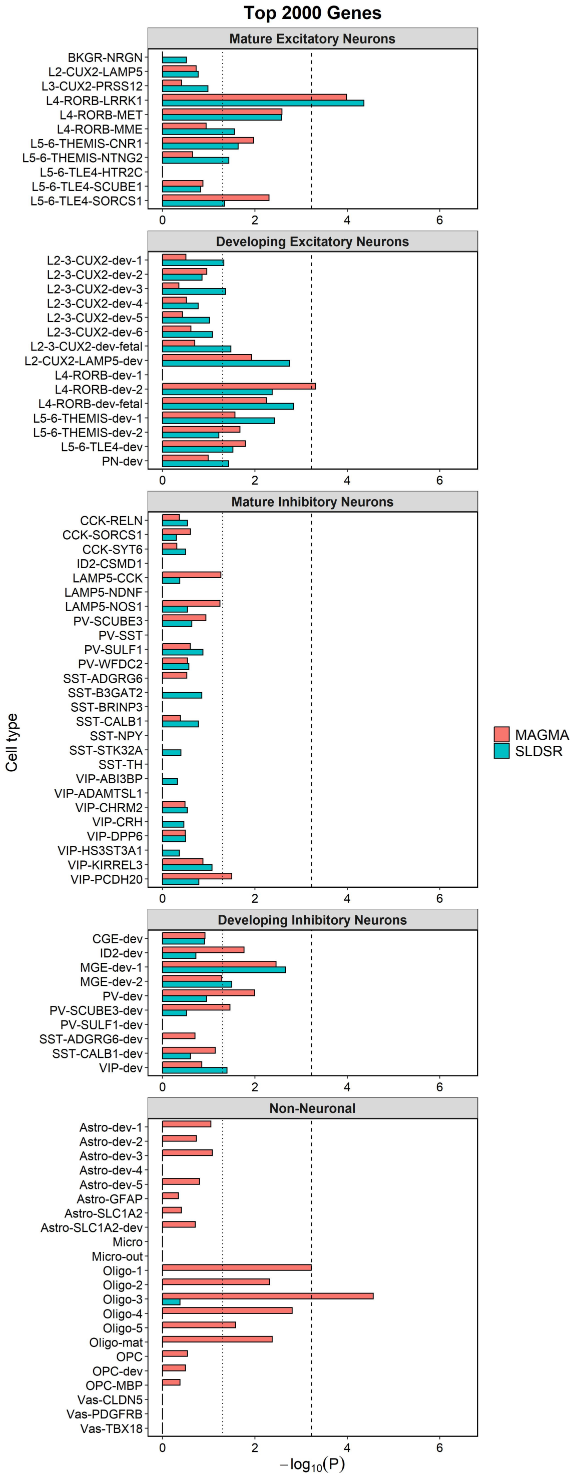

Supplementary Figure S1. MAGMA and SLDSR -log$_{10}$ *P*-values for enrichment of schizophrenia common variant liability within the 2000 genes with highest expression specificity for each prefrontal cortex (PFC) cell population. The dotted line shows the nominal (*P* < 0.05) significance threshold, and the dashed line shows the Bonferroni-corrected *P*-value threshold for the 84 cell populations tested ($P < 5.95 \times 10^{-4}$). Cell populations are labelled in accordance with Herring et al. (PMID: 36318921) on the basis of cell markers. L = layer; dev = developing cells; Astro = astrocytes; Micro = microglia; Oligo = oligodendrocytes; OPC = oligodendrocyte precursor cell; Vas = vascular cell. For a full list of abbreviations, see Supplementary Table S16.

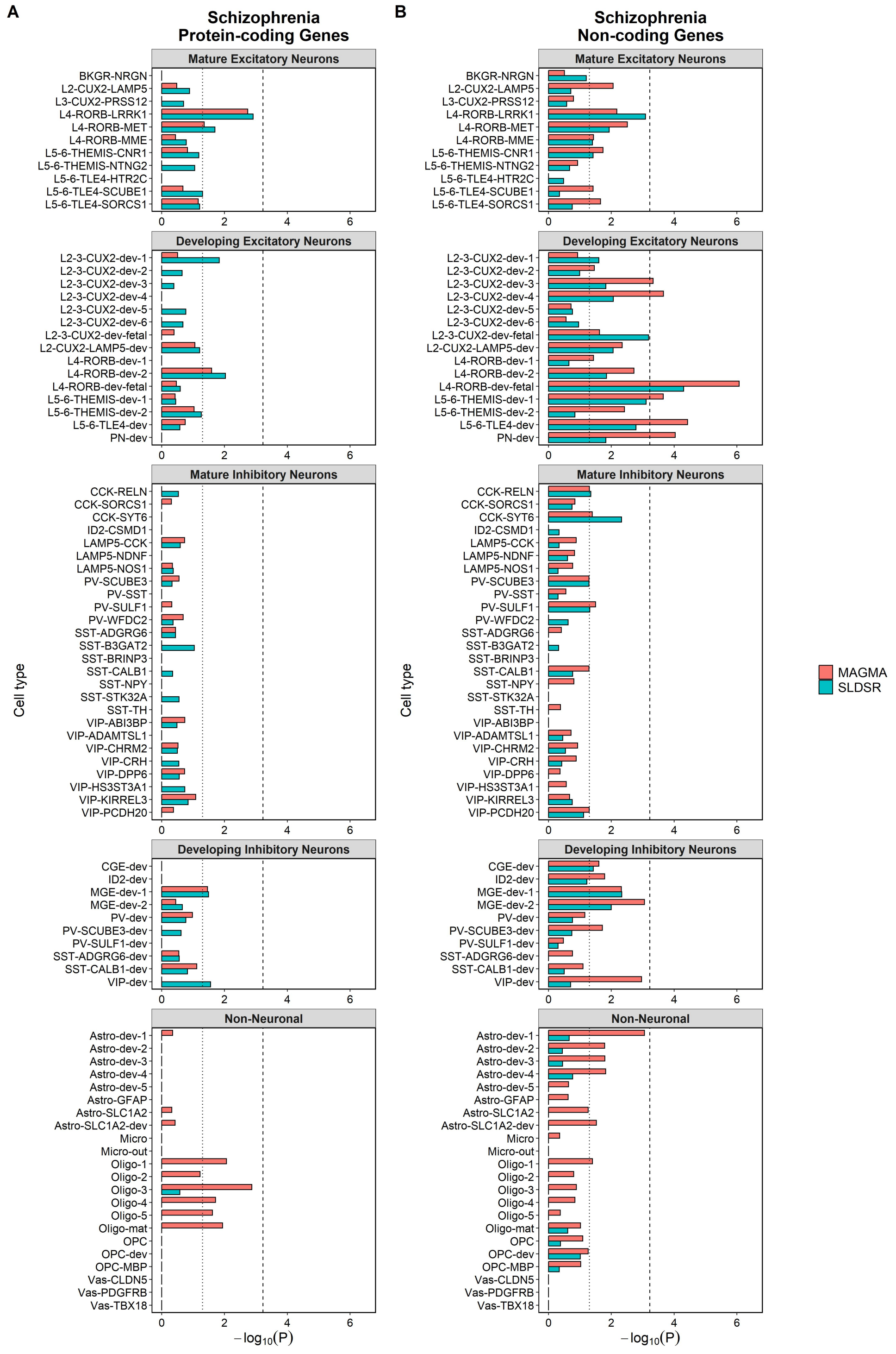

Supplementary Figure S2. MAGMA and SLDSR -$\log_{10}$ *P*-values for enrichment of schizophrenia common variant liability within A) protein coding genes and B) non-coding genes in the top expression specificity decile of each PFC cell population. The dotted line shows the nominal ($P < 0.05$) significance threshold, and the dashed line shows the Bonferroni-corrected *P*-value threshold for the 84 cell populations tested ($P < 5.95 \times 10^{-4}$). Cell populations are labelled in accordance with Herring et al. (PMID: 36318921) on the basis of cell markers. L = layer; dev = developing cells; Astro = astrocytes; Micro = microglia; Oligo = oligodendrocytes; OPC = oligodendrocyte precursor cell; Vas = vascular cell. For a full list of abbreviations, see Supplementary Table S16.

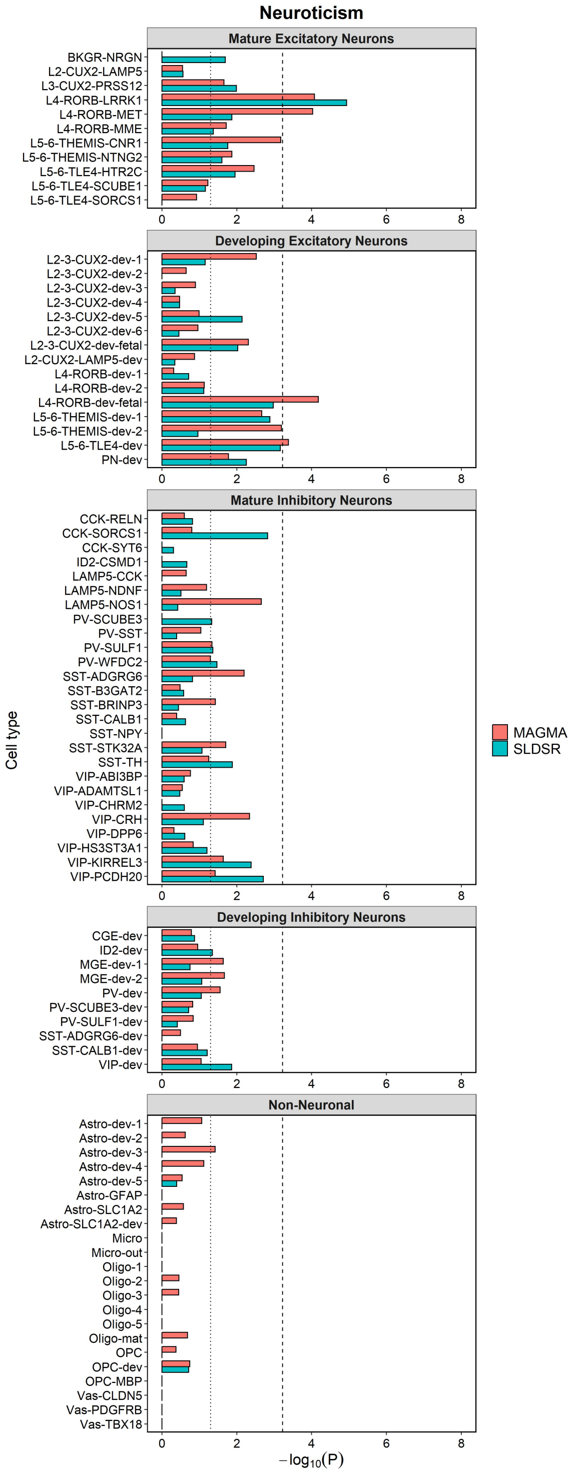

Supplementary Figure S3. MAGMA and SLDSR -log$_{10}$ *P*-values for enrichment of common variants associated with neuroticism in the top decile of expression specificity for each cell population of the prefrontal cortex. The dotted line shows the nominal (*P* < 0.05) significance threshold, and the dashed line shows the Bonferroni-corrected *P*-value threshold for the 84 cell populations tested ($P < 5.95 \times 10^{-4}$). Cell populations are labelled in accordance with Herring et al. (PMID: 36318921) on the basis of cell markers. L = layer; dev = developing cells; Astro = astrocytes; Micro = microglia; Oligo = oligodendrocytes; OPC = oligodendrocyte precursor cell; Vas = vascular cell. For a full list of abbreviations, see Supplementary Table S16.

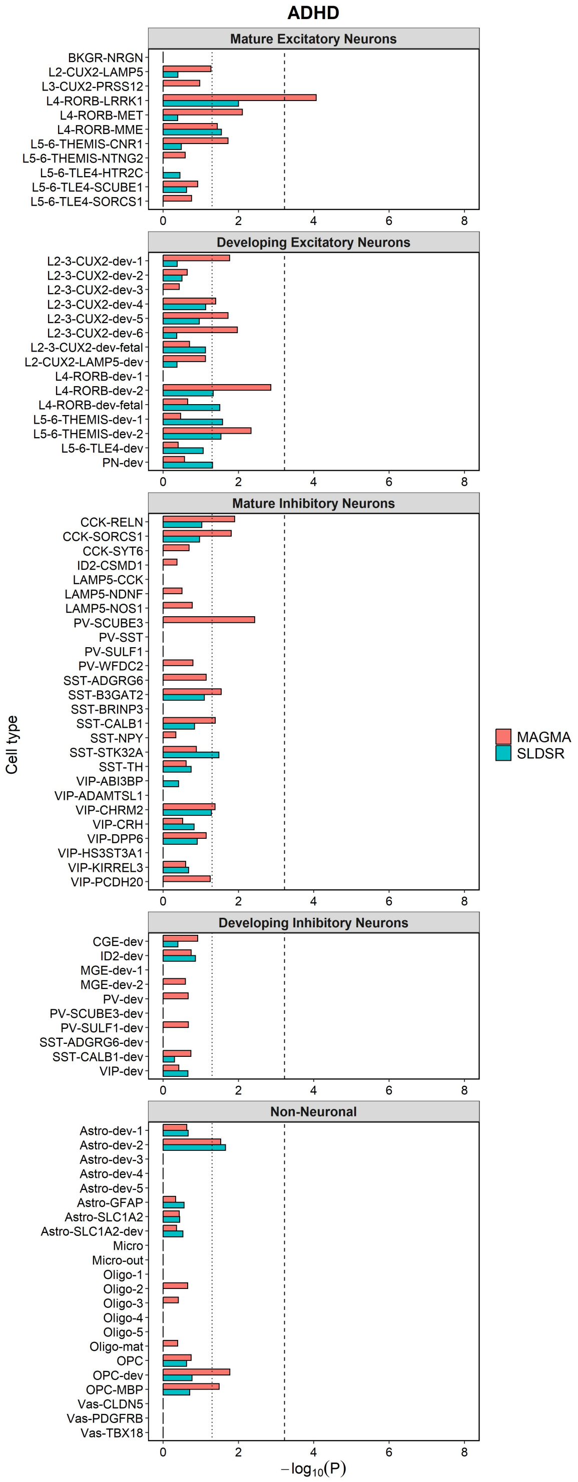

Supplementary Figure S4. MAGMA and SLDSR -log$_{10}$ *P*-values for enrichment of common variants associated with ADHD in the top decile of expression specificity for each cell population of the prefrontal cortex. The dotted line shows the nominal ($P < 0.05$) significance threshold, and the dashed line shows the Bonferroni-corrected *P*-value threshold for the 84 cell populations tested ($P < 5.95 \times 10^{-4}$). Cell populations are labelled in accordance with Herring et al. (PMID: 36318921) on the basis of cell markers. L = layer; dev = developing cells; Astro = astrocytes; Micro = microglia; Oligo = oligodendrocytes; OPC = oligodendrocyte precursor cell; Vas = vascular cell. For a full list of abbreviations, see Supplementary Table S16.

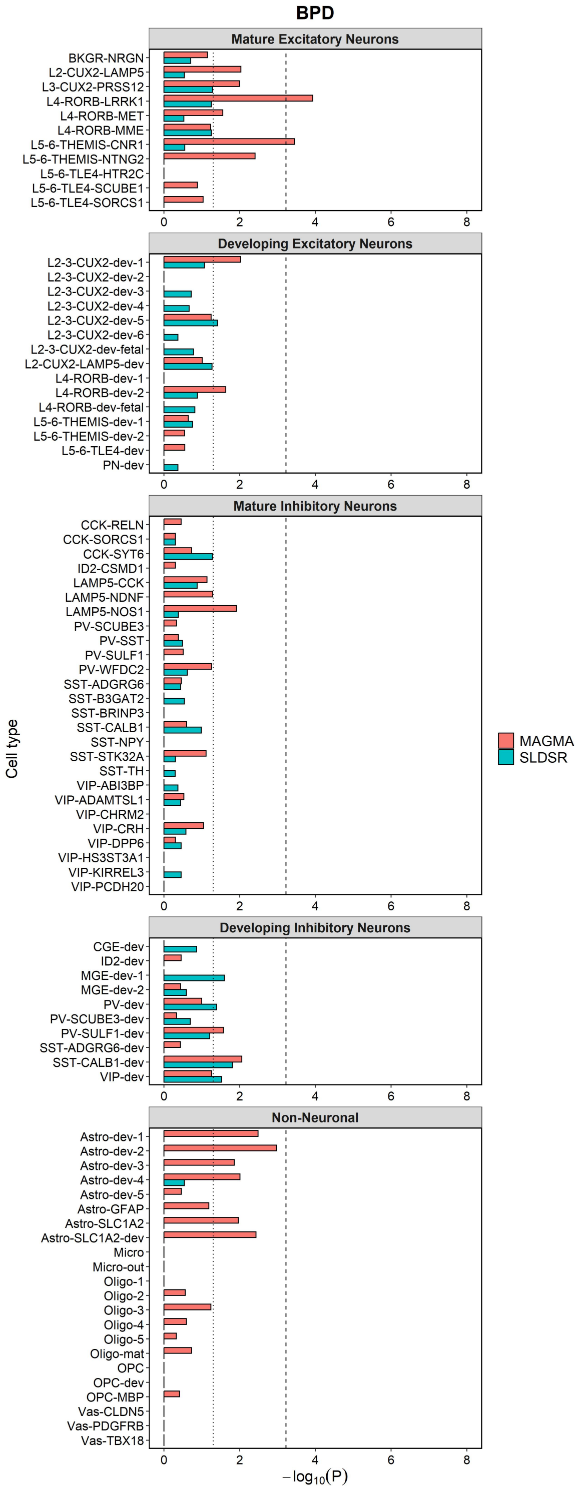

Supplementary Figure S5. MAGMA and SLDSR -log$_{10}$ *P*-values for enrichment of common variants associated with bipolar disorder in the top decile of expression specificity for each cell population of the prefrontal cortex. The dotted line shows the nominal ($P < 0.05$) significance threshold, and the dashed line shows the Bonferroni-corrected *P*-value threshold for the 84 cell populations tested ($P < 5.95 \times 10^{-4}$). Cell populations are labelled in accordance with Herring et al. (PMID: 36318921) on the basis of cell markers. L = layer; dev = developing cells; Astro = astrocytes; Micro = microglia; Oligo = oligodendrocytes; OPC = oligodendrocyte precursor cell; Vas = vascular cell. For a full list of abbreviations, see Supplementary Table S16.

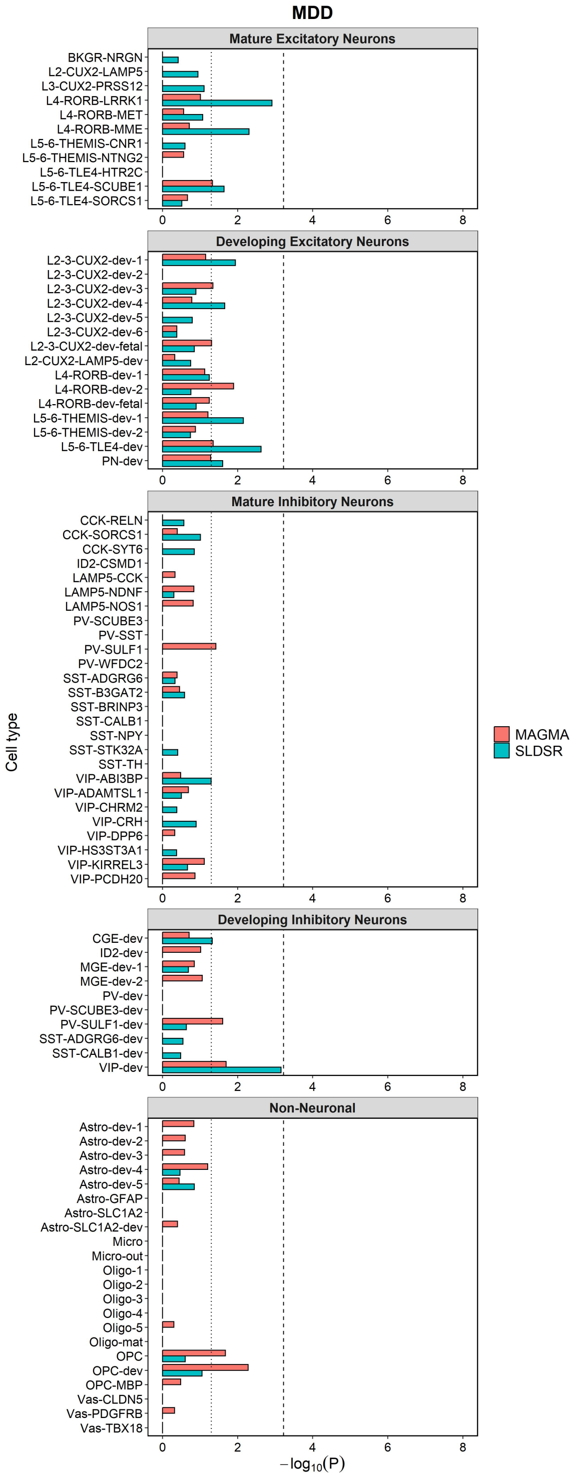

Supplementary Figure S6. MAGMA and SLDSR -log$_{10}$ *P*-values for enrichment of common variants associated with major depressive disorder in the top decile of expression specificity for each cell population of the prefrontal cortex. The dotted line shows the nominal ($P < 0.05$) significance threshold, and the dashed line shows the Bonferroni-corrected *P*-value threshold for the 84 cell populations tested ($P < 5.95 \times 10^{-4}$). Cell populations are labelled in accordance with Herring et al. (PMID: 36318921) on the basis of cell markers. L = layer; dev = developing cells; Astro = astrocytes; Micro = microglia; Oligo = oligodendrocytes; OPC = oligodendrocyte precursor cell; Vas = vascular cell. For a full list of abbreviations, see Supplementary Table S16.

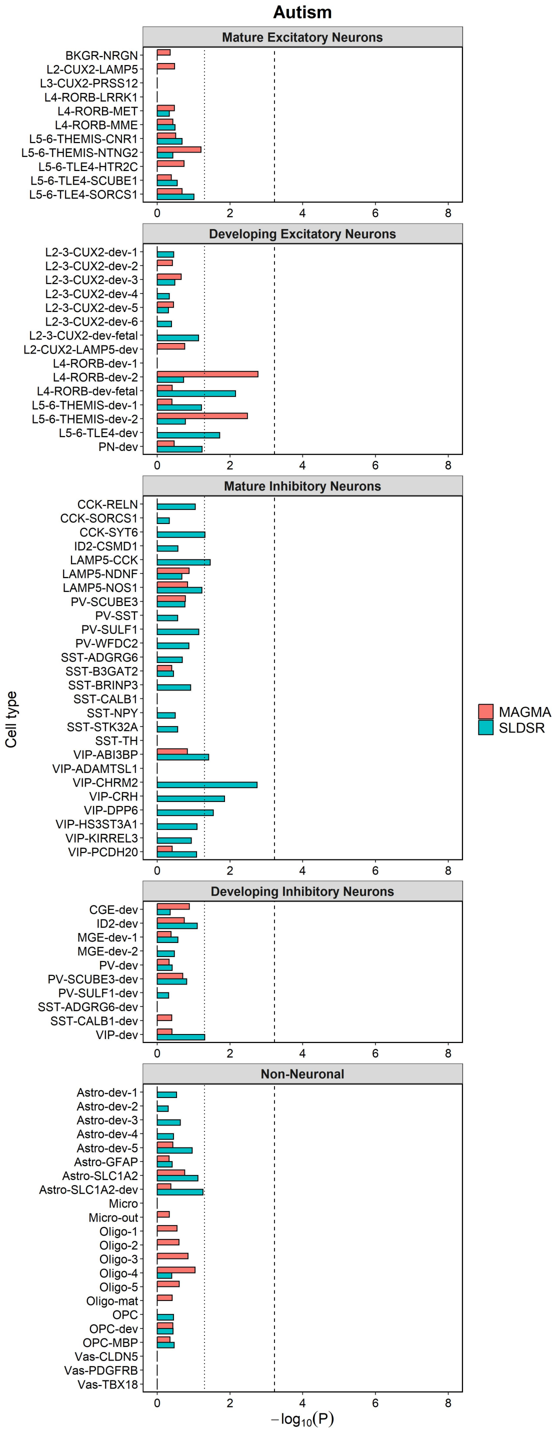

Supplementary Figure S7. MAGMA and SLDSR -log$_{10}$ *P*-values for enrichment of common variants associated with autism in the top decile of expression specificity for each cell population of the prefrontal cortex. The dotted line shows the nominal (*P* < 0.05) significance threshold, and the dashed line shows the Bonferroni-corrected *P*-value threshold for the 84 cell populations tested (*P* < 5.95 X 10^{-4}). Cell populations are labelled in accordance with Herring et al. (PMID: 36318921) on the basis of cell markers. L = layer; dev = developing cells; Astro = astrocytes; Micro = microglia; Oligo = oligodendrocytes; OPC = oligodendrocyte precursor cell; Vas = vascular cell. For a full list of abbreviations, see Supplementary Table S16.

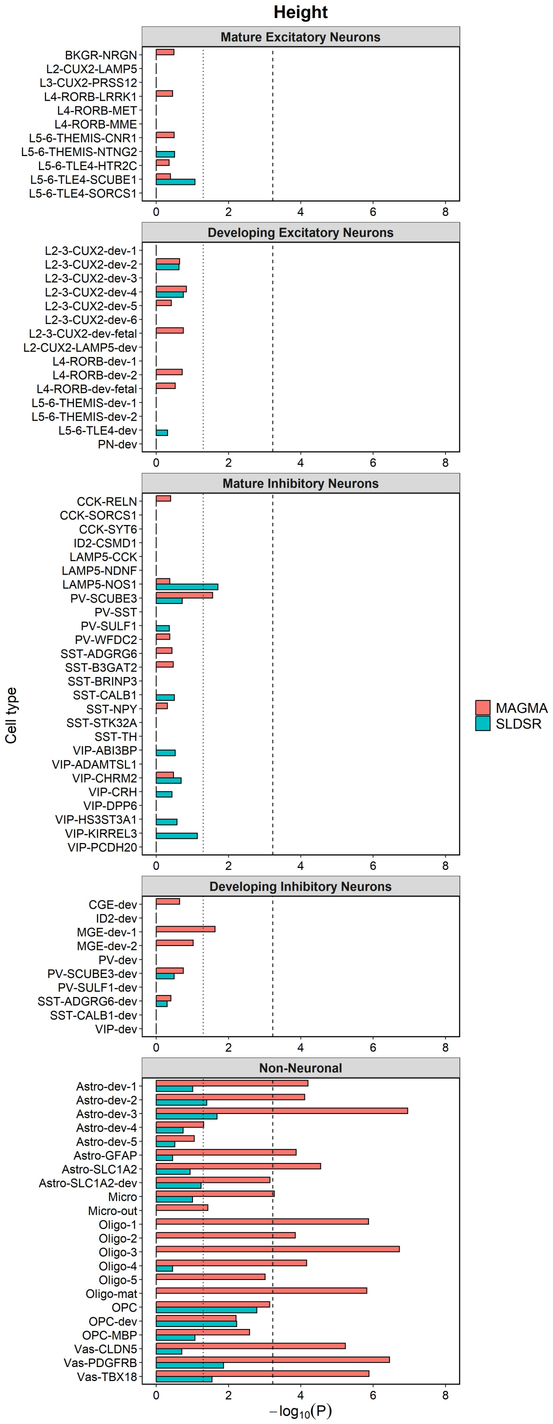

Supplementary Figure S8. MAGMA and SLDSR -log$_{10}$ *P*-values for enrichment of common variants associated with height in the top decile of expression specificity for each cell population of the prefrontal cortex. The dotted line shows the nominal ($P < 0.05$) significance threshold, and the dashed line shows the Bonferroni-corrected *P*-value threshold for the 84 cell populations tested ($P < 5.95 \times 10^{-4}$). Cell populations are labelled in accordance with Herring et al (PMID: 36318921) on the basis of cell markers. L = layer; dev = developing cells; Astro = astrocytes; Micro = microglia; Oligo = oligodendrocytes; OPC = oligodendrocyte precursor cell; Vas = vascular cell. For a full list of abbreviations, see Supplementary Table S16.

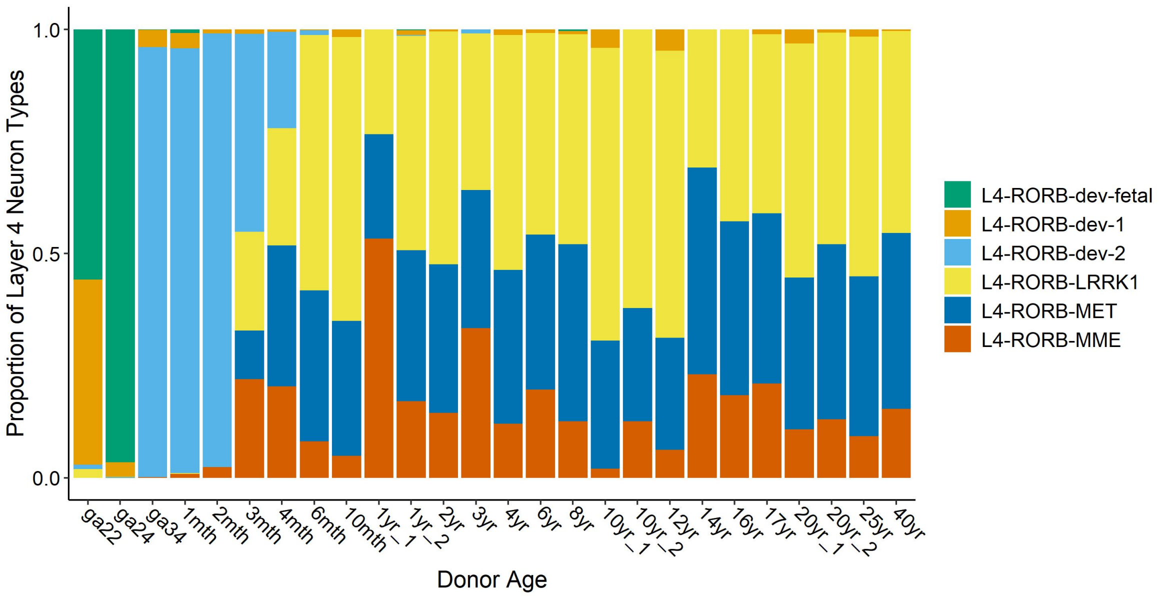

Supplementary Figure S9. Subpopulations of RORB+ layer 4 glutamatergic neurons as a proportion of the total number of RORB+ layer 4 glutamatergic neurons across development of the prefrontal cortex. Mature RORB+ populations comprise L4-RORB-LRRK1, L4-RORB-MET and L4-RORB-MME. GA = gestational age. Data and cell labels taken from Herring et al. (PMID: 36318921).

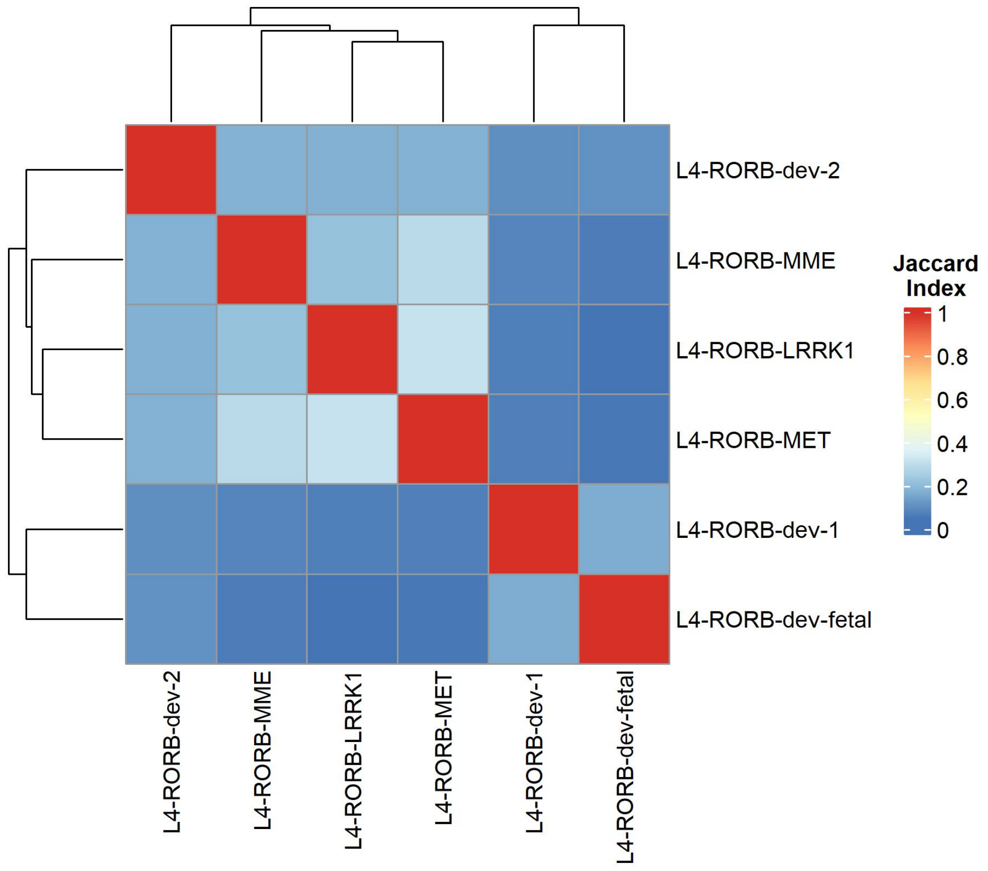

Supplementary Figure S10. Jaccard index between gene sets representing the top decile of expression specificity for each RORB+ neuron population of the prefrontal cortex. Dev = developing. Mature RORB+ populations comprise L4-RORB-LRRK1, L4-RORB-MET and L4-RORB-MME. The maximum Jaccard index between the top decile of expression specificity gene sets for RORB+ cell populations is 0.31.

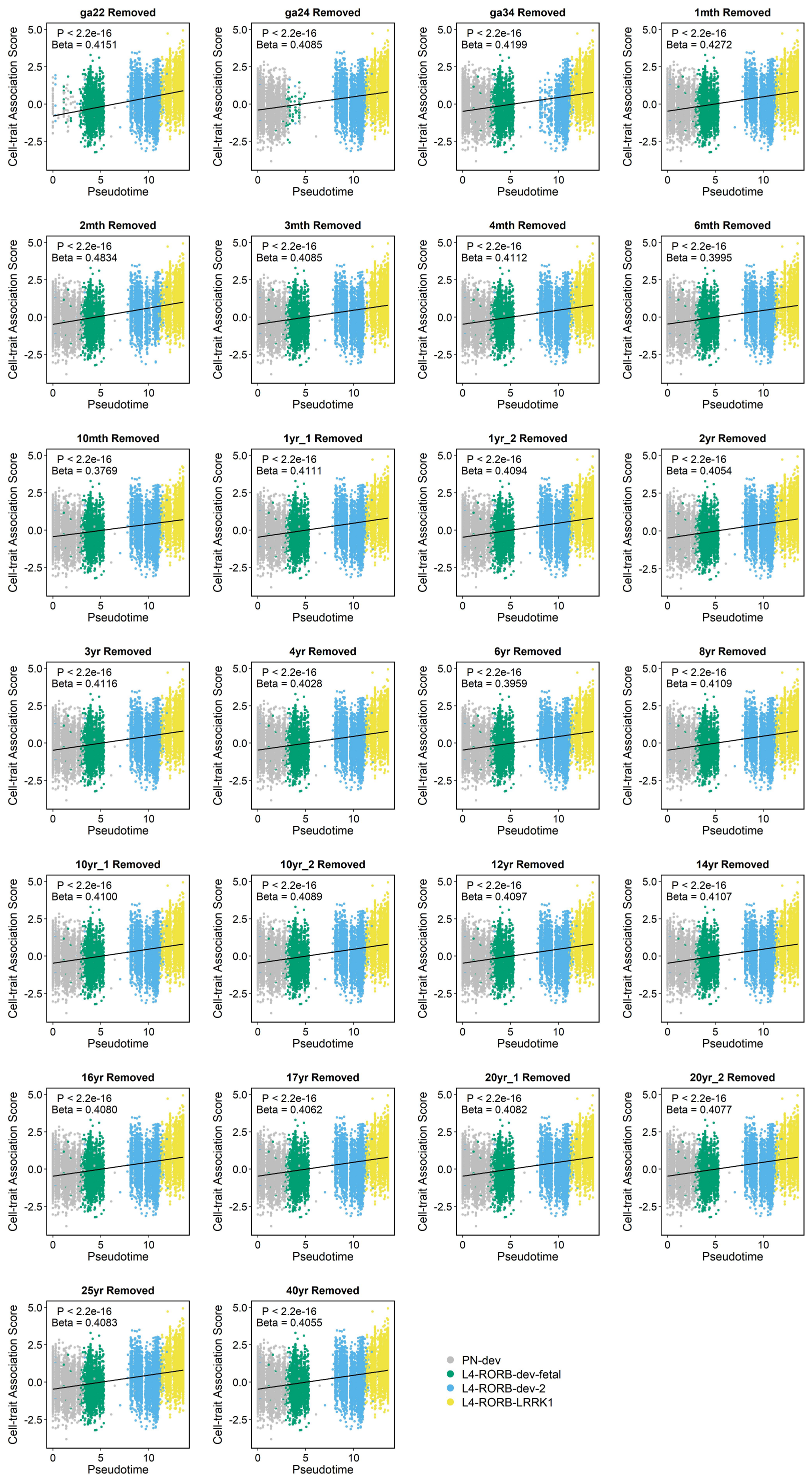

Supplementary Figure S11. Leave-one-out tests for relationship between cell-trait (schizophrenia) association scores and pseudotime values along the developmental trajectory of L4–RORB-LRRK1 neurons. Cell-trait association scores were calculated based on the relationship between MAGMA gene analysis schizophrenia *P*-values and the expression level of each gene detected in each individual cell following the method of Shulman and Elkon (PMID: 34194671). The significant increase in cell-trait association scores along the L4-RORB-LRRK1 developmental trajectory was maintained when cells belonging to each donor (age of excluded donor indicated at top of each panel) were removed (all $P < 2.2 \times 10^{-16}$).
